# Protecting the Airway and the Physician: Lessons from 214 Cases of Endotracheal Intubation Litigation

**DOI:** 10.1155/2022/8209644

**Published:** 2022-10-18

**Authors:** Jean Daniel Eloy, Anna A. Pashkova, Molly Amin, Christy Anthony, Daisy Munoz, Yuriy Gubenko, Shivani Patel, Anna Korban, Andrea Perales, Peter F. Svider, Jean Anderson Eloy

**Affiliations:** ^1^Department of Anesthesiology, Rutgers New Jersey Medical School, Newark, NJ, USA; ^2^Department of Otolaryngology–Head and Neck Surgery, Wayne State University School of Medicine, Detroit, MI, USA; ^**3**^ Department of Otolaryngology–Head & Neck Surgery, Rutgers New Jersey Medical School, Newark, NJ, USA; ^**4**^ Department of Neurological Surgery, University of Medicine and Dentistry of NJ, New Jersey Medical School, Newark, NJ, USA; ^**5**^ Center for Skull Base and Pituitary Surgery, University of Medicine and Dentistry of NJ, New Jersey Medical School, Newark, NJ, USA

## Abstract

**Objective:**

Medicolegal examination of an intervention as common as endotracheal intubation may be valuable to physicians in many specialties. Our objectives were to comprehensively detail the factors raised in litigation to better educate physicians on strategies for minimizing liability and augmenting patient safety.

**Methods:**

Publicly available court records were searched for pertinent litigation. Ultimately, 214 jury verdict and settlement reports were examined for various factors, including outcome, award, geographic location, defendant specialty, setting in which an injury occurred, patient demographics, and other causes of malpractice.

**Results:**

Ninety-two cases (43.0%) were resolved in the defendant's favor, with the remaining cases resulting in out-of-court settlement or a plaintiff's verdict. Payments from these cases were considerable, averaging $2.5 M. The most frequent physician defendants were anesthesiologists (59.8%) and emergency-physicians (19.2%), although other specialties were well represented. The most common setting of injury was the operating room (45.3%). Common factors included sustaining permanent deficits (89.2%), death (50.5%), and anoxic brain injury (37.4%). Injuries occurring in labor and delivery mostly involved newborns and had among the highest awards.

**Conclusions:**

Litigation involves injuries sustained in numerous settings. The most common factors present included sustaining permanent deficits, including anoxic brain injury. The presence of this latter injury increased the likelihood of a case being resolved with payment. Finally, deficits in informed consent were noted in numerous cases, stressing the importance of a clear process in which the physician explains specific risks (such as those detailed in this analysis), benefits, and alternatives.

## 1. Introduction

Endotracheal intubation is used in a wide variety of settings for airway management. Particularly in critically ill patients, the potential for adverse consequences may be considerable [1], ranging from cuff pressure injuries to anoxic brain injury and death. Difficult intubation, especially in emergent situations, has also previously been associated with increased morbidity and mortality. [2] Long-term sequelae such as tracheal stenosis are also possible [3–6]. Newer technologies such as fiberoptic intubation and video laryngoscopy have been increasingly used over traditional direct laryngoscopy and may potentially facilitate appropriate airway management by improving vocal cord visualization during intubation [7].

Costs associated with medical malpractice litigation have been rising over the past three decades and may account for up to $10 billion extra in health expenditures annually [8–13]. Previous analyses have noted a number of reasons that influence the outcome and initiation of litigation. Although many of these factors may be specific to iatrogenic injuries and adverse outcomes, recurrent themes have been noted in the literature. An appreciable portion of these analyses have noted deficits in the informed consent process, having to undergo additional reparative procedures as a result of a complication and undergoing unnecessary interventions as major factors affecting litigation. [4, 13–25] Additionally, experiencing an irreversible deficit may certainly play a role in the initiation of litigation [23].

The potential for devastating consequences from injuries that affect quality of life makes this a subject of interest for physicians practicing a variety of specialties and may be of particular importance to anesthesiologists. Anesthesiology has long been considered a specialty at high risk for medical malpractice claims. In the early 1980s, it was shown that 11% of total dollars paid for patient injury were caused by anesthetic related complications, despite anesthesiologists only accounting for 3% of all physicians insured (source = Lee). This caused professional liability insurance premiums to soar. As a result, the American Society of Anesthesiologists (ASA) Closed Claims Project was established in 1984 to improve patient safety and prevent anesthetic injury, which would then decrease claims and payments and drive down the cost of insurance premiums. Analysis of data obtained from closed claims in the ASA Closed Claims Project database has resulted in valuable discoveries that have since helped change the practice of anesthesiology to improve patient safety (source = Cheney). Thus, the utility of closed claims analysis utilizing the ASA Closed Claims Project as well as other databases may continue to yield important information in the future. Respiratory system adverse events, including those related to endotracheal intubation, represent the most common mechanism leading to anesthesia malpractice claims and account for a large proportion of claims for death and brain damage (source = Peterson). After an exhaustive literature search, the authors were unable to identify any previous analyses of malpractice litigation related solely to endotracheal intubation. Our objectives were to comprehensively detail factors raised in jury verdicts and settlement reports, including outcomes and awards, defendants involved, settings of injury, and other alleged causes of malpractice. Our hope is that by describing the findings from these cases and characterizing the medicolegal aspects of endotracheal intubation, this information can be used to better educate the physician on strategies for minimizing liability, increasing communication with patients regarding expectations and risks, and consequently, further improving the patient safety.

## 2. Materials and Methods

The terms “medical malpractice” and “intubation” were entered into the advanced search function of the Westlaw legal database (Thomson Reuters, New York, NY). This database contains publicly available federal and state court records. Data collection patterns vary by jurisdiction; therefore, they were not standardized in the database. Various commercial vendors supply court records to Westlaw; while a minority of jurisdictions may only contain court reports voluntarily submitted by attorneys involved, [26, 27] most locales include involuntarily submitted records as well, designating parties involved in these cases with terms such as “confidential,” “John/Jane Doe,” and “anonymous.” [28] The laypersons involved in submitting such data do not have any medical knowledge. Although many out-of-court settlements may potentially not progress far enough to the point of inclusion in these publicly available court records, this legal database contains the vast majority of cases that have made it to trial, and has thus been of value in previous comprehensive analyses of various medicolegal topics, including (but not limited to) hearing loss [26], facial plastic surgery [13], tracheal stenosis [4], corticosteroid use [27], cranial nerve injury [24], otolaryngology litigation [22, 29], facial nerve injury [17], iatrogenic orbital injury [23], and cerebrospinal fluid leaks [16].

**Figure 1:**
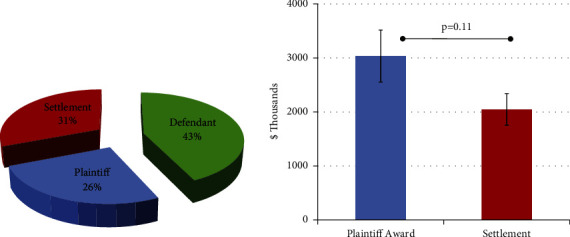
The consort diagram for the total cases reviewed.

Out of 460 jury verdict and settlement reports that were initially found using the search terms, cases were excluded for the following reasons: intubation was not related to the reason for malpractice litigation (145), duplicate cases (18), litigation was initiated due to failure to intubate rather than adverse sequelae related to an intubation (i.e., intubation was not performed) (83) Figure 1. The remaining 214 jury verdicts and settlement reports were examined for various factors, including outcome, award, geographic location, defendant specialty, setting in which an injury from intubation occurred, surgical procedure, clinical manifestations of injury, patient demographics, and causes of malpractice. The reviewers completed a standardized form that recorded the previously mentioned factors. Since most of the cases had multiple factors listed in litigation, all the factors listed per case were included when reviewing the data. The data collection were completed in April 2013.

### 2.1. Statistical Analysis

Comparison of continuous variables was performed using Mann-Whitney *U*-tests and Kruskal–Wallis tests, while comparison of categorical data was performed using Chi-Square analysis as appropriate, with the threshold for significance set at *p* < 0.05.

## 3. Results

**Figure 2:**
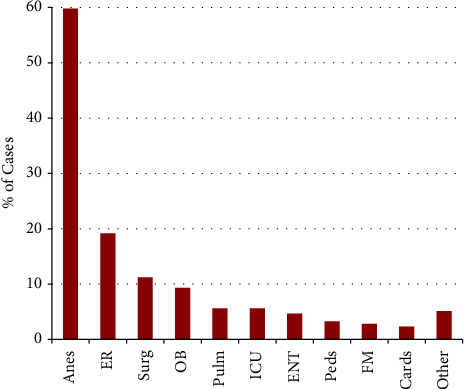
Outcome and mean payments. Standard error bars represent standard error of mean.

Out of 214 jury verdict and settlement reports included in this analysis, 92 (43.0%) were resolved with a defendant verdict, with the remaining cases split between a plaintiff verdict and an out-of-court settlement (Figure 2). Proceedings resolved with a settlement or jury award resulted in considerable payments (mean payment = $2.51 M + - 275,869 standard error of mean); mean plaintiff awards were higher than out-of-court settlements, although this relationship did not reach statistical significance (*p* = 0.11) (Figure 2). In this analysis, 58.4% of plaintiffs were women and 41.6% were men. The mean plaintiff age was 38.9 years (+-1.99 SEM).

**Figure 3:**
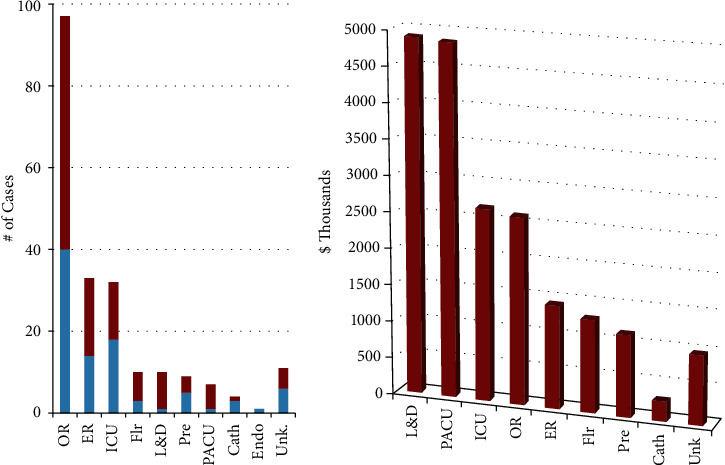
Physician defendant specialty. Anes = anesthesiology, ER = emergency medicine, surg = general surgery, OB = obstetrics and gynecology, pulm = pulmonology, ICU = intensive care unit (unspecified), ENT = otolaryngology, peds = pediatrics, FM = family medicine, cards = cardiology.

**Figure 4:**
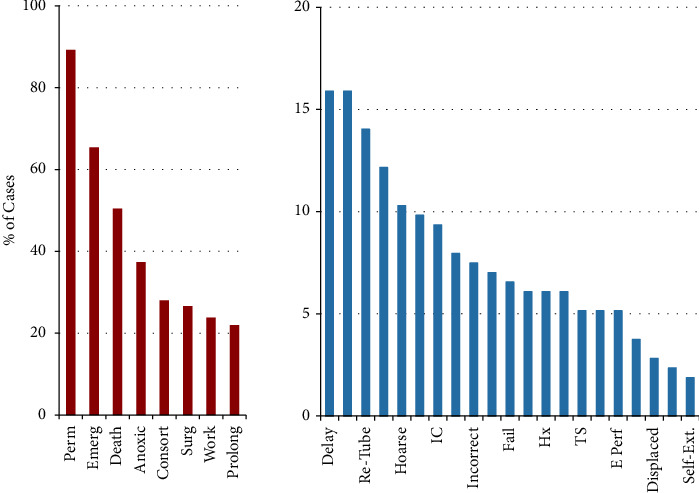
(a) Setting of alleged cause of malpractice, red (top) portion of bars represent cases resolved with payment (either out-of-court settlement or jury award), blue (bottom) portion of bars represent defendant verdicts. (b) Mean payment organized by setting. OR = operating room, ER = emergency room, ICU = intensive care unit, Flr = floor, L&*D* = labor and delivery, Pre = prehospital, PACU = anesthesia recovery unit, Cath = cardiac catheterization lab, Endo = endoscopy suite, Unk. = unclear location.

**Figure 5:**
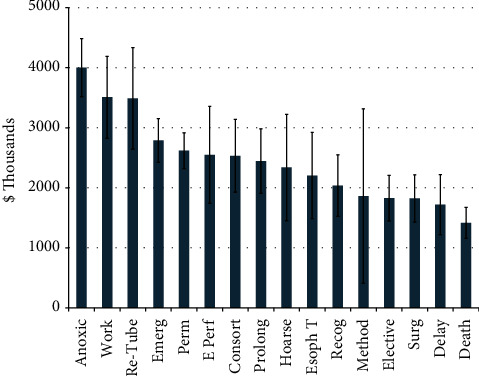
Most cited factors in malpractice litigation. Perm = permanent injury, Emerg = emergent intubation, Anoxic = anoxic brain injury, Consort = loss of consortium, Surg = required reparative surgery, Work = employment/income affected, Prolong = prolonged intubation, Delay = delayed intubation, Esoph *T* = esophageal intubation, Re-tube = reintubation, Recog = delayed recognition of complication, Hoarse = hoarseness, Method = other method should have been used, IC = perceived deficit in informed consent, Trauma = traumatic intubation, Incorrect = “incorrect intubation” (unspecified), Asp = aspiration, Fail = failed intubation, Early = premature extubation, Hx = neglected medical history, Unn. = unnecessary intubation, TS = tracheal stenosis, Teeth = injury to teeth, E Perf = esophageal perforation, VC = vocal cord injury, Displaced = displaced endotracheal tube, Size = incorrect ET tube size, Self-Ext. = self-extubation by patient.

**Figure 6:**
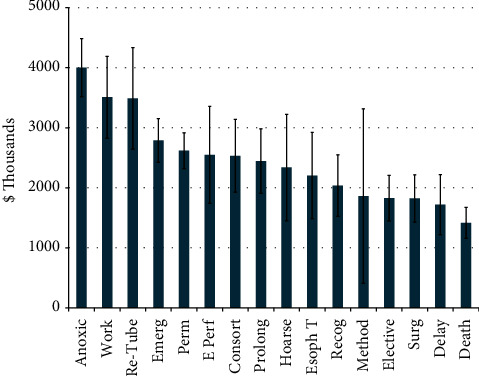
Mean payments of cases containing the most common factors cited in litigation. Abbreviations same as those in the previous figure.

Anesthesiologists were the most named defendants (59.8%), followed by emergency physicians (19.2%), general surgeons (11.2%), obstetricians and gynecologists (9.3%), pulmonologists and other intensivists (5.6% each), and otolaryngologists (4.7%) (Figure 3). Consequently, the most common setting in which an intubation injury was to occur was in the operating room (45.3% of all cases), within which 58.8% of cases were resolved with a payment (Figure 4), while the next most common settings were the emergency room (16.3%) and intensive care unit (15.0%). Payment averages varied by setting (Kruskal–Wallis, *p* < 0.05) (Figure 4). The most common factors present in litigation included permanent deficit because of an injury (89.2%), undergoing an emergent intubation (65.4%), death (50.5%), and anoxic brain injury (37.4%) (Figure 5). Other commonly cited factors are also noted in Figure 5. Although differences in outcome were noted with the presence of many of these factors, anoxic brain injury was the only factor that was statistically more likely to be resolved with payment (67.5% of cases) than in cases in which this factor was not noted (50.7%) (*p* = 0.02) (Table 1). Other situations, including esophageal perforation, self-extubation, delay in recognizing a complication, delayed intubation, and requiring reparative surgery, had a higher proportion of cases resolved with a payment when these factors were present, although these differences did not reach statistical significance (*p*-values >0.05) (Table 1). The most common factors were examined both if present and the likelihood of payout versus not present and the likelihood of payment (Table 1). Payment amounts in cases resolved with an out-of-court settlement or jury award varied among the most common factors cited in litigation (Kruskal–Wallis Test, *p* < 0.005) (Figure 6). The geographic locations of cases included in this analysis showed that the most populous states generated the most cases (Table 2).

## 4. Discussion

The use of endotracheal intubation has increased over the past three decades with the rapid proliferation of minimally invasive procedures that minimize perioperative risks for certain populations, allowing an increasing number of patients to undergo a wide variety of interventions. For such a common technique such as endotracheal intubation, which may be utilized in both emergent and nonemergent situations, a comprehensive examination of factors integral in malpractice litigation may serve as a valuable resource for physicians. Such an analysis may provide strategies for minimizing liability and augmenting patient safety. Several devastating consequences may be associated with the use of endotracheal intubation, making this a potential target of malpractice litigation.

Although very generally patients alleged a permanent deficit in nearly 90% of malpractice cases included in this analysis (Figure 5), specific consequences such as death, anoxic brain injury, hoarseness, aspiration, tracheal stenosis, and esophageal injury were noted in many cases. Additionally, nonspecific issues, such as loss of consortium, requiring additional reparative surgery, having employment/income affected, and a delay in recognizing a complication, were noted in a substantial portion of cases. The presence of anoxic brain injury (37.4% of litigation) increased the likelihood of a case being resolved with payment (Table 1); additionally, payments for such an injury were among the highest of all factors, averaging over $4 million (Figure 6). Although the additional likelihood of an outcome resulting in payment did not reach statistical significance for other factors or did not differ from cases in which individual factors were not raised, the issues raised in Table 1 are still important factors in initiating litigation. It should be stressed that most costs directly associated with litigation arise through legal representation fees, the cost of expert witnesses, and court payments, rather than coming from jury awards or settlements [12, 30]. This suggests that just because something was not shown to increase the likelihood of a payment does not mean it should be disregarded. Additionally, when payments were made, they were considerable, as the mean payment in this analysis exceeded $2.5 M, and many of the more common factors brought up in litigation averaged over $1M (Figure 6).

The occurrence of an unexpected or adverse outcome is not sufficient for a jury to compel a defendant to pay damages for medical negligence. Several factors, well described in the medicolegal literature and explicitly outlined in state and federal laws, must be present, including the presence of a duty to act; an obvious deviation from this duty that results in departure from a standard of care; the occurrence of an adverse or harmful event; and a demonstration that a physician's actions directly caused an adverse event [4, 30, 31]. Consequently, there has been a concerted attempt in recent years to educate jurors and the lay public about this idea that simply experiencing a harmful event is not the same thing as being a victim of medical malpractice [31].

Anesthesiologists were the most common physician defendants named in this analysis, named in 59.8% of cases (Figure 3). Despite this finding, the figures detailed in this study may be of interest to physicians practicing a wide variety of specialties, including emergency medicine, surgery, obstetrics and gynecology, critical care, and otolaryngology. Several possibilities may be offered for this observation. As reported, litigation is stemmed from the use of endotracheal intubation in a variety of settings (Figure 4). Another potential explanation may be that plaintiffs usually cast a wide net while naming physician defendants in the hope that this would make these entities more amenable to a sizeable out-of-court settlement, partially obviating the time and financial investment necessary in malpractice litigation that progresses to trial [32, 33].

As noted in Figure 4, occurrences of malpractice during labor and delivery had among the highest resulting payments ($4.9 M, +-159,00 SEM), although this finding did not reach statistical significance upon comparison with the next 3 most common settings, including cases in the anesthesia recovery unit, cases in the intensive care unit, and malpractice occurring in the operating room (*p* > 0.05). One possibility for this finding is the fact that most cases (7 of 9) in labor and delivery involved the newborn rather than the mother. These newborns were considerably younger than all cases in non-L&D situations (median 40.7, *p* < 0.005). The substantial economic cost of improper intubation of a newborn along with the required lifelong care for such patients sustaining a permanent injury likely contributed to this observed figure.

Deficits in informed consent were cited in approximately 10% of cases in this analysis (Figure 5), substantially lower than several previous analyses of malpractice litigation [13, 19, 24, 28]. Despite the paucity of specific informed consent allegations, patients in many of these cases may have benefitted from a thorough informed consent process that explicitly explained risks, benefits, and alternatives, as such patient-physician communication would increase understanding of any procedures and allow a patient to make a more informed choice [22, 24, 34, 35]. For example, specifically listing specific risks (as appropriate) noted in Figure 5 would certainly achieve this goal. Finally, although only 20 jury reports specifically noted the mention of informed consent, there are likely many more cases where an unexpected outcome contributed to a patient's decision to initiate litigation. Despite the well-described importance of a comprehensive informed consent process, a significant proportion of cases in this analysis dealt with emergent situations (65.4%, Figure 5), likely making opportunities for a thorough informed consent process challenging.

Advances in technology have provided practitioners with numerous options for airway management. Consequently, newer innovations may not have been around and in widespread use for the entire 25-year duration of this analysis. It should be noted that the data from the Westlaw database are very heterogeneous, and few cases mentioned specific techniques such as fiberoptic intubation and video laryngoscopy, thus ensuring such a discussion would have been inconclusive. Like innovations increasing a physician's therapeutic repertoire, the field of medical malpractice is also constantly evolving secondary to changes in the law and the dynamic nature of litigation. As a result, while the findings from this analysis comprehensively detail litigation regarding endotracheal intubation over the past 25 years up through the present, a future analysis including sources that detail the use of these newer technologies may be a valuable adjunct to our present study.

In addition to the heterogeneous nature of the Westlaw database, it should be stressed that out-of-court settlements may be under-represented, making Westlaw more valuable for a discussion of specific risk factors present in litigation progressing far enough for inclusion into public records rather than an analysis of the overall prevalence of initiated or explored litigation regarding injuries from endotracheal intubation. The comprehensive details involved in these jury verdict and settlement reports, however, provide a rich source of information from which issues integral to determining legal responsibility can be examined. Consequently, this database has shown its unique value in prior analyses of important medical topics through numerous studies [4, 13, 16, 17, 21–24, 26, 27, 29, 36–39] _ENREF_12 [17].

## 5. Conclusions

With the considerable costs that medical errors and subsequent malpractice litigation contribute to healthcare spending in the United States, medicolegal examination of an intervention as common as endotracheal intubation may be valuable to physicians in many specialties for identifying strategies to minimize liability and enhance patient safety. Out-of-court settlements and jury awards were substantial in the 57% of cases in this analysis resulting in payments, averaging over $2.5 million, suggesting that characterization of factors involved in litigation may be important in better understanding how to minimize the financial and health impact of these cases. Although anesthesiologists were the most commonly named defendants and the operating room was the most common setting in malpractice, our findings emphasize that litigation regarding intubation injuries still affects practitioners in a wide variety of settings and specialties. The most common factors present in litigation included sustaining a permanent deficit, including anoxic brain injury. The presence of this latter injury increased the likelihood of a case being resolved with payment and was among the injuries with the highest average payments. Finally, deficits in informed consent were noted in multiple cases, stressing the importance of a clear process in which the physician explains specific risks (such as those named in this analysis), benefits, and alternatives.

## Figures and Tables

**Table 1:** Prevalence of factors mentioned in cases organized by outcome.

Factor	% Resolved with payment when factor present	% Resolved with payment when factor is not present
Esophageal perforation◉	77.8%	56.1%
Self-extubation◉	75.0%	56.7%
Delay in recognizing/Treating complication	73.1%	54.8%
Anoxic brain injury^*∗∗*^	67.5%	50.7%
Delayed intubation	64.7%	55.6%
Neglected past medical history	61.5%	56.7%
Additional surgery required for repair	61.4%	55.4%
Incorrect ET tube size◉	56.2%	57.1%
Employment/Income affected	58.8%	56.4%
Emergent intubation	58.9%	54.1%
Esophageal intubation	58.1%	56.8%
Permanent deficit	57.6%	52.2%
Prolonged attempt	57.4%	56.9%
Incorrect intubation (unspecified)	56.3%	57.1%
Death	55.6%	58.5%
Loss of consortium	55.0%	57.8%
Displaced tube◉	50.0%	57.2%
Traumatic intubation	47.1%	57.9%
Aspiration	46.7%	57.8%
Unnecessary	46.2%	57.7%
Other method should have been used	42.9%	58.5%
Deficit in informed consent	40.0%	58.8%
Hoarseness	36.4%	59.4%
Vocal cord damage◉	25.0%	58.1%
Tracheal stenosis◉	18.2%	59.1%

Cases “resolved with payment” refers to both settlements and plaintiff verdicts. “◉” = Occurred in <5% of cases in this analysis. ^*∗∗*^ = Statistically significant difference between case outcome when factor was present versus when factor was not present, as measured by chi-square test with two-tailed *p*-value <0.05.

**Table 2:** Case jurisdiction.

State	^#^ of cases
CA	35
FL	28
MI	20
NY	18
OH	16
MA	15
PA	10
TX	10
IL	8
MO	6
VA	6
MD	5
WA	5
GA	4
CT	3
IA	3
IN	3
NV	3
AZ	2
DC	2
KS	2
OK	2
Other	8

## Data Availability

We used publicly available data from the Westlaw legal database (Thomson Reuters, New York, NY). This database contains publicly available federal and state court records.
